# TCONS_00012883 promotes proliferation and metastasis via DDX3/YY1/MMP1/PI3K‐AKT axis in colorectal cancer

**DOI:** 10.1002/ctm2.211

**Published:** 2020-10-14

**Authors:** Peng Yang, Jie Li, Chaofan Peng, Yuqian Tan, Ranran Chen, Wen Peng, Qiou Gu, Jiahui Zhou, Lu Wang, Junwei Tang, Yifei Feng, Yueming Sun

**Affiliations:** ^1^ The First School of Clinical Medicine Nanjing Medical University Nanjing China; ^2^ Department of General Surgery The First Affiliated Hospital of Nanjing Medical University Nanjing China

**Keywords:** colorectal cancer, DDX3, MMP1, TCONS_00012883, YY1

## Abstract

**Background:**

Long noncoding RNAs (lncRNAs) have emerged as key regulators in multiple cancers, including colorectal cancer (CRC). However, the biological functions and molecular mechanisms underlying most lncRNAs in CRC remain largely unknown.

**Methods:**

A novel lncRNA (TCONS_00012883) was identified using RNA sequencing. The level of TCONS_00012883 expression in CRC was analyzed by qRT‐PCR. The biological functions of TCONS_00012883 in CRC were investigated by a series of *in vitro* and *in vivo* experiments: CCK8, colony formation, EdU, flow cytometric assays, transwell assays, and mouse xenograft. The molecular mechanisms of TCONS_00012883 were demonstrated by RNA pulldown, mass spectrometry analysis, RIP, coimmunoprecipitation, RNA sequencing, chromatin immunoprecipitation, and rescue experiments.

**Results:**

Elevated expression of TCONS_00012883 was confirmed in CRC and positively associated with a poor prognosis. Functionally, gain‐ and loss‐of‐function assays indicated that TCONS_00012883 promoted proliferation and metastasis of CRC cell lines *in vitro* and *in vivo*. Mechanistically, RNA pulldown and mass spectrometry analysis showed that DEAD‐box helicase 3 (DDX3) was the protein partner of TCONS_00012883. Furthermore, RNA sequencing assay revealed that matrix metallopeptidase 1 (MMP1) was the downstream of TCONS_00012883. Intriguingly, we found that transcription factor (YY1) could serve as a bridge between TCONS_00012883, DDX3, and MMP1.

**Conclusions:**

TCONS_00012883 significantly promoted CRC progression via the DDX3/YY1/MMP1 axis, and thus, may act as a major role in diagnosis and therapy of CRC.

## Graphical abstract

Graphical Headlights: 1. This study provides the first evidence that a novel lncRNA TCONS_00012883 promotes proliferation and metastasis in colorectal cancer. 2. TCONS_00012883 mediates the transactivation of YY1 through interacting with DDX3. 3. TCONS_00012883 regulates MMP1 expression.

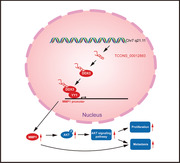

**Abbreviations**
CCK‐8: Cell Counting Kit‐8
CRC: colorectal cancer
DDX3: DEAD‐box helicase 3
EdU: 5‐Ethynyl‐2′‐deoxyuridine
ESR1: estrogen receptor 1
LncRNAs: long noncoding RNAs
MMP1: matrix metallopeptidase 1
OS: overall survival rate
PFS: progression‐free survival rate
qRT‐PCR: quantitative real‐time PCR
TFs: transcription factors
TNM: tumor‐node‐metastasis
TP53: tumor protein p53
YY1: Yin Yang 1

## 1 BACKGROUND

Colorectal cancer (CRC) is one of the most common malignant tumors of digestive tract, and its incidence rate and mortality rate are all on the rise.^1^ Despite the substantial development in early diagnosis and therapeutic approaches, CRC continues to have high incidence and mortality rates.^2^ Most advanced patients have a poor prognosis because of advanced recurrence and metastasis.^3^, ^4^, ^5^ One of the main reasons is that the mechanisms underlying CRC remain unclear. Thus, there is an urgent need to investigate the unknown mechanisms underlying the progression of CRC and develop novel diagnostic markers and more efficient targeted therapies.

Noncoding RNAs (ncRNAs) have recently attracted the attention of a large group of researchers.^6^, ^7^ As an emerging category of regulatory molecules, ncRNAs have been verified to control cancer progression. For instance, miR‐127 prodrug suppresses the growth and metastatic potential of breast cancer.^8^ Similarly, circ_0001361 could regulate bladder cancer invasion and metastasis.^9^ Among these ncRNAs, lncRNAs are a class of transcripts > 200 nucleotides (nt) in size.^10^ LncRNAs were originally considered to be “junk” of genome transcription, as a by‐product of transcription with no biological functions.^11^, ^12^ Recently, an abundance of studies have shown that lncRNAs could participate in multiple biological processes, such as chromatin modification, transcription activation, stem cell differentiation, and immune responses.^11^, ^12^, ^13^ Accumulating evidence confirms that lncRNAs play a major role in the regulation of cancer progression.^14^, ^15^ Generally, lncRNA functions are exerted through cis/trans gene regulation, and interaction with proteins, such as RNA‐binding protein (RBP) FUBP3,^16^ or by playing the role of ceRNAs for miRNAs.^17^, ^18^ In recent years, the interaction between lncRNAs and RBPs has a great progress. For instance, lnc02023 regulates the stability of PTEN.^19^ LncRNA ELIT‐1 facilitates TGF‐β/Smad signaling through interacting with Smad3.^20^ Although researches on lncRNAs have progressed rapidly, the functions of most lncRNAs are still unclear. Therefore, it is essential to explore the functions of lncRNAs in CRC progression.

In the current study, a novel lncRNA (TCONS_00012883) was identified using RNA sequencing (RNA‐seq) for the first time. TCONS_00012883 was located at 7q21.11 (1965 nt in RNA size). Literature review revealed that the pattern of expression and biological functions of TCONS_00012883 in cancer have not been established. The coding potential assessment tool (CPAT^21^) predicted that TCONS_00012883 has a very low coding potential. Unfortunately, the function of TCONS_00012883 and its regulatory mechanisms in cancer progression remained elusive. Thus, this study attempted to determine the roles of TCONS_00012883 in CRC. We showed that TCONS_00012883 was markedly upregulated in CRC and associated with a poor prognosis. Gain‐ and loss‐of‐function assays confirmed that TCONS_00012883 could be an oncogene and that it promoted CRC cell proliferation and metastasis. The related mechanisms indicated that TCONS_00012883 regulated matrix metallopeptidase 1 (MMP1) expression by interacting with DEAD‐box helicase 3 (DDX3) to mediate the transactivation of Yin‐Yang 1 (YY1). Our study demonstrated the regulatory molecular mechanism of TCONS_00012883 in CRC progression and confirmed that TCONS_00012883 could act as a major role in diagnosis and therapy of CRC.

## 2 MATERIALS AND METHODS

### 2.1 Human specimens and cell culture

Two hundred fresh tumor and matched normal tissues were collected from CRC patients in the First Affiliated Hospital of Nanjing Medical University between 2014 and 2018. None of the patients received chemotherapy nor radiotherapy preoperatively before surgery. All samples were frozen and stored at ‐80°C conservation. The study was approved by the Institutional Ethical Board of our hospital.

DLD‐1, LoVo, HCT 116, HT‐29, and Caco‐2 and a normal epithelial colon cell (NCM460) were obtained from the ATCC. DLD‐1, LoVo, and Caco‐2 cells were cultured in RPMI‐1640 medium (HyClone, Logan, UT, USA), while HCT 116 and HT‐29 were cultured in McCoy’5A medium. All medium were supplemented with 10% fetal bovine serum. The cells were cultured at 37°C in a moist incubator stabilized at 5% CO_2_.

### 2.2 RNA extraction and quantitative real‐time PCR

TRIzol reagent (Invitrogen, Carlsbad, CA) was applied in the isolation of RNA as described previously.^22^ Total RNA (0.5 μg) was further reverse transcribed into cDNA through the HiScript RT Mix (Vazyme, Jiangsu, China). SYBR Green Kit (TaKaRa Biotechnology, Dalian, China) was used for quantitative real‐time PCR (qRT‐PCR). GAPDH was used as internal controls. The sequences of primers are shown in Table S6. The raw data are shown in Table S8.

### 2.3 RNA and protein isolation of nuclear and cytoplasmic fractions

DLD‐1, LoVo, HCT 116, and HT‐29 cells were separated into cytoplasmic and nuclear fractions using a PARIS kit (#AM1921; ThermoFisher). RNA and protein were isolated from each fraction according to the protocol. The levels of TCONS_00012883, U6, and GAPDH or β‐actin RNA were analyzed using qRT‐PCR.

### 2.4 RNA interference and plasmids

The lentivirus containing shRNAs targeting TCONS_00012883 were synthesized by Obio (Shanghai, China). The full length of TCONS_00012883 synthesized by Obio was subcloned into the lentivirus vector. The shRNAs targeting DDX3, YY1, TP53, ESR1, and MMP1 and the corresponding negative controls (sh‐NC) were synthesized by RiboBio (Guangzhou, China). MMP1 overexpression plasmid was obtained from Obio. Lipofectamine 3000 (Invitrogen) was used for the transfection of shRNAs and plasmid vectors. The transfection efficiency was confirmed through qRT‐PCR. The sequences are listed in Table S6.

### 2.5 Western blot analysis and antibodies

Western blot (WB) was performed as reported previously.^22^ The primary antibodies are shown in Table S7.

### 2.6 Cell proliferation assay

The Cell Counting Kit‐8 (CCK‐8; Beyotime, Shanghai, China) was used to detect cell proliferation.^23^

For the colony formation assay, the treated cells were seeded onto six‐well plates as described previously.^23^

### 2.7 5‐Ethynyl‐2′‐deoxyuridine assay

The EdU Kit (Beyotime) was applied to detect the cell proliferation.^23^ Proliferation was analyzed using the mean number of cells in three fields for each sample.

### 2.8 Cell cycle and apoptosis analysis

The treated cells were digested and centrifuged at 1500 rpm for 5 min. After twice‐washing, the cells were fixed with ethanol at ‐20°C overnight. Then, they were stained with DNA staining solution (Beyotime) for 30 min in the dark at room temperature. The percentages of cells were analyzed using BD FACSCanto II (BD Biosciences, San Jose, CA, USA).

The apoptosis assay was performed using an Annexin V‐APC /7‐AAD Apoptosis Detection Kit (KeyGEN, Jiangsu, China). The treated cells were incubated with 0.5 mM of H_2_O_2_ for 4 h to stimulate apoptosis and then the cells were digested and centrifuged at 1500 rpm for 5 min. Annexin V‐APC and 7‐AAD staining solutions were used according to the protcol. The apoptotic rate was analyzed using BD FACSCanto II.

### 2.9 Transwell assay

The transwell assay was performed as reported previously.^24^ Three random fields were selected and counted using a microscopy.

### 2.10 RNA sequencing assay

Total RNA analyzed with an Agilent 2200 TapeStation and those samples that passed quality inspection (OD260/280 ≥ 1.5, OD260/230 ≥ 1.0, complete agarose gel electrophoresis strip, RNA integrity number ≥ 7, and 28S/16S ≥ 1.0) were constructed with a starting amount of 1 μg of total RNA to generate the sequencing libraries (RiboBio). The main steps were as follows: mRNA capture and fragmentation; first‐ and second‐strand cDNA synthesis with random hexamer primers; repair the ends of the double‐stranded cDNA fragments; add A to the 3′ end of the DNA fragments; adaptor ligation, fragment selection, and purification; and PCR amplification and purification. The cDNA was then sequenced in a HiSeq 2000 system on Pair End (Illumina, San Diego, CA, USA).

### 2.11 Fluorescence in situ hybridization

A FISH Kit (RiboBio) was applied to detect the location of lncRNA. Cells were fixed for 10 min. Then, the fixed cells were then permeabilized for 5 min. The treated cells were incubated with prehybridization buffer for 30 min. Fluorescence in situ hybridization (FISH) probes were mixed with preheated hybridization buffer and added into the cells overnight at 37°C under dark conditions. DAPI was used as a nuclear stain. Results were analyzed using a confocal fluorescence microscopy.

### 2.12 RNA pulldown assay and mass spectrometry analysis

TCONS_00012883 and antisense RNA were transcribed *in vitro* with Taq Master Mix (Vazyme, Jiangsu, China) and the products were labeled with T7 Biotin by using T7 Enzyme mix and Biotin RNA Labelling Mix (RiboBio). Cell lysates were incubated with biotinylated RNAs and 50 μL of magnetic beads (Invitrogen) for 60 min. The complex was boiled in loading buffer and the resolved protein was analyzed through WB analysis or mass spectrometry analysis (BGI Shenzhen, Guangdong, China).

### 2.13 RIP and coimmunoprecipitation assay

An RIP Kit (Millipore, Burlington, MA, USA) was applied in this study. In brief, 50 μL of a magnetic beads suspension was washed and resuspended in 100 μL of RIP wash buffer. Then, 5 μg of anti‐DDX3 antibodies were added into each tube and incubated with rotation for 30 min. One hundred microliter lysates were added to each beads‐antibody complex in RIP immunoprecipitation buffer and all the tubes were incubated while rotating overnight at 4°C. The purified RNA was analyzed using RT‐PCR or qRT‐PCR.

An IP/Co‐IP Kit (#88828, ThermoFisher) and Co‐IP Kit (#26149, ThermoFisher) were used to determine the interaction between DDX3 and YY1. For DDX3 immunoprecipitation, DDX3 antibody was immobilized on AminoLink Plus Coupling Resin, and then incubated with cell lysates overnight at 4°C. After elution of the immunoprecipitation products, the products were boiled for 10 min with 5X Lane Marker Sample Buffer for the next analysis. For YY1 immunoprecipitation, the whole lysates were incubated with the beads‐antibody complex. After the products were washed with lysis buffer, they were boiled for 10 min with 1 × SDS loading buffer for the next analysis. The primary antibodies used are listed in Table S7.

### 2.14 Immunofluorescence

For immunofluorescence colocalization, cells were cultured on a confocal laser dish. After incubation with FISH probes, as described above, the cells were blocked with an immunostaining blocking solution (P0102; Beyotime) for 60 min, and then incubated with DDX3 or YY1 overnight. The cells were incubated with fluorescently labeled secondary antibody for 60 min in the dark. DAPI was used to visualize the nuclei. The primary antibodies used are shown in Table S7.

### 2.15 Chromatin immunoprecipitation

A ChIP Kit (CST, #56383, Danvers, MA, USA) was used to determine if YY1 bound to the MMP1 promoter. Cells (4 × 10^6^) were crosslinked in 1% formaldehyde for 10 min, and then incubated with glycine solution for 5 min. Then, the cells were cracked with 500 μL of chromatin immunoprecipitation (ChIP) sonication cell lysis buffer twice and 500 μL of ChIP sonication nuclear lysis buffer. After shearing by sonication, 90% chromatin fragment was concentrated between 200 and 1000 bp. Fifty microliters of sheared crosslinked chromatin were incubated with a beads‐antibody complex with rotation. The purified DNA was analyzed using qRT‐PCR. The primer sequences are shown in Table S6. The sequence motif was predicted using JASPAR database.^25^

### 2.16 Immunohistochemistry

Immunohistochemistry (IHC) was performed as previously described.^23^ The primary antibodies used are listed in Table S7.

### 2.17 Animal models

All animal experiments were approved by the Committee on the Ethics of Animal Experiments of Nanjing Medical University. Five‐week‐old BALB/c nude male mice were used for the xenograft model and tumor metastasis assay. For tumorigenicity studies, DLD‐1 cells stably transfected with TCONS_00012883 and control cells were separately implanted into the left and right groins of the mice. The tumor volumes and weights were measured every 5 days. Twenty‐five days after the injection, the xenograft tumors were dissected and weighed for IHC. For the tumor metastasis assay, the treated cells were injected into the splenic artery through the sup‐spleen. After 4 weeks, the liver tissues were detected through H&E staining.

### 2.18 Statistical analysis

Each experiment was repeated three times. The results are shown as the mean ± standard deviation. SPSS software 19.0 was used for the statistical analyses, including Student's *t*‐test (two‐tailed), Pearson's correlation analysis, Kaplan‐Meier analysis, and the log‐rank test. The significance threshold was set at 0.05 for in each test.

## 3 RESULTS

### 3.1 TCONS_00012883 is upregulated in CRC and associated with a poor prognosis

**FIGURE 1:**
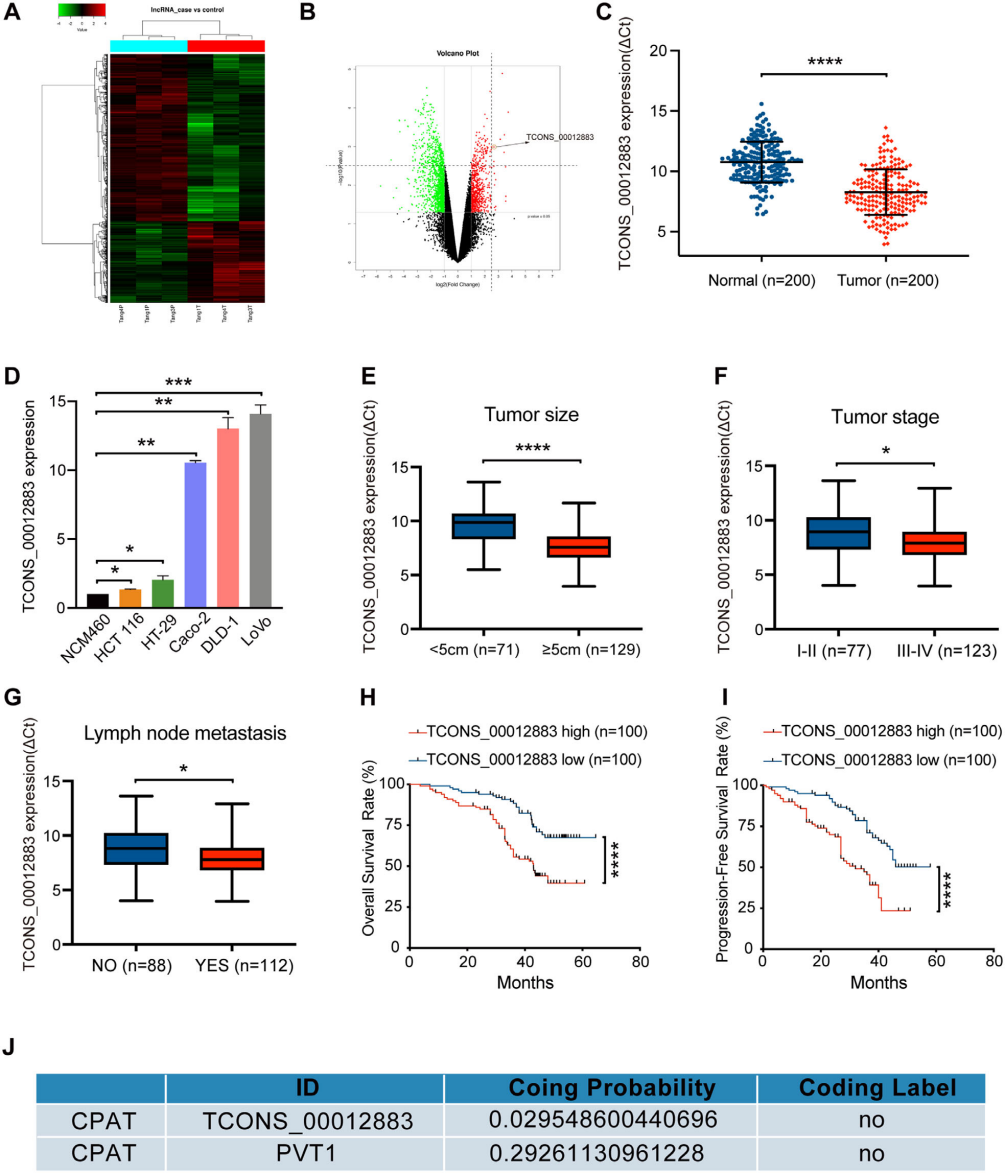
LncRNA TCONS_00012883 is upregulated in CRC and associated with a poor prognosis. A, Heat map with hierarchical clustering of the differentially expressed lncRNAs between colorectal cancer samples and normal samples (with fold change > 2 and *P* < .05). B, LncRNAs (with fold change > 2 and *P* < .05) plotted as a volcano plot. C, Relative expression of LncRNA TCONS_00012883 detected by qRT‐PCR in 200 pairs colorectal cancer tissues and matched normal tissues. ΔCt = Ct (TCONS_00012883) – Ct (GAPDH). Results are presented as Δ cycle threshold (ΔCt) in tumor tissues and normal tissues. Paired *t*‐test was used for the statistical analyses. D‐F, Relative expression of TCONS_00012883 in CRC with different tumor size, tumor stage, and lymph node metastasis. ΔCt = Ct (TCONS_00012883) – Ct (GAPDH). Results are presented as Δ cycle threshold (ΔCt) in tumor tissues. G, H, Kaplan‐Meier plots of the OS and PFS of CRC patients with high (n = 100) and low (n = 100) levels of TCONS_00012883. I, qRT‐PCR analysis of the relative expression of TCONS_00012883 in five CRC cell lines and the immortalized normal epithelial colon cell NCM460. J, Predicted coding potential of TCONS_00012883 in CPAT, known lncRNA PVT1 as positive control. Data are presented as the mean ± SD. *P < .05, **P < .01 and ***P < .001, ****P < .0001

RNA‐seq was performed to identify the differentially expressed lncRNAs involved in CRC (Figure 1A, B). The first seven overexpressed lncRNAs with log_2_(Fold Change) ≥ 2.5 and ‐log_10_ (*P* value) ≥ 2.5 were shortlisted. As shown in Figure S1A, PVT1, TRPM2‐AS, and TCONS_00012883 were most significantly higher in 24 pairs of tumor tissues than normal tissues. There were many reports about PVT1 and TRPM2‐AS, while none about TCONS_00012883. So, we selected this novel gene, and believed that TCONS_00012883 was more worthy of our in‐depth study. In Figure 1C, TCONS_00012883 expression was higher in 200 pairs of tumor tissues than in matched normal samples. In addition, TCONS_00012883 expression was markedly higher in CRC cell lines (DLD‐1, LoVo, Caco‐2, HCT 116, and HT‐29) compared with that in normal epithelial colon cell NCM460 (Figure 1D). FISH also confirmed that TCONS_00012883 was upregulated in other gastrointestinal cancer, such as gastric cancer and pancreatic cancer (Figure S5). A total of 200 patients were separated into two groups by using the median level of expression as the cutoff value (n = 100 > median; n = 100 < median). Table S1 shows that the expression of TCONS_00012883 was highly correlated with the tumor size (*P* = .002), TNM staging system (*P* = .005), tumor stage (*P* = .014), lymph node metastasis (*P* = .011), and distant metastasis (*P* = .030). The level of TCONS_00012883 expression was also analyzed in different clinical subgroups. The expression was higher in tumor samples with larger tumor size (size ≥ 5 cm), advanced tumor stage (stages III and IV), and lymph node metastasis (Figure 1E‐G). However, it was not increased in patients with distant metastasis (Figure S2A). Kaplan‐Meier survival analysis indicated that patients with higher TCONS_00012883 levels had a shorter overall survival (OS) and progression‐free survival (PFS) than patients with lower levels of TCONS_00012883 (Figure 1H, I). The coding potential assessment tool (CPAT) predicted that TCONS_00012883 has a very low coding potential (known lncRNA PVT1 as a positive control; Figure 1J). Collectively, these clinical data suggest that the upregulated expression of TCONS_00012883 is related to CRC proliferation and metastasis.

### 3.2 TCONS_00012883 promotes CRC cell proliferation and metastasis *in vitro*

**FIGURE 2:**
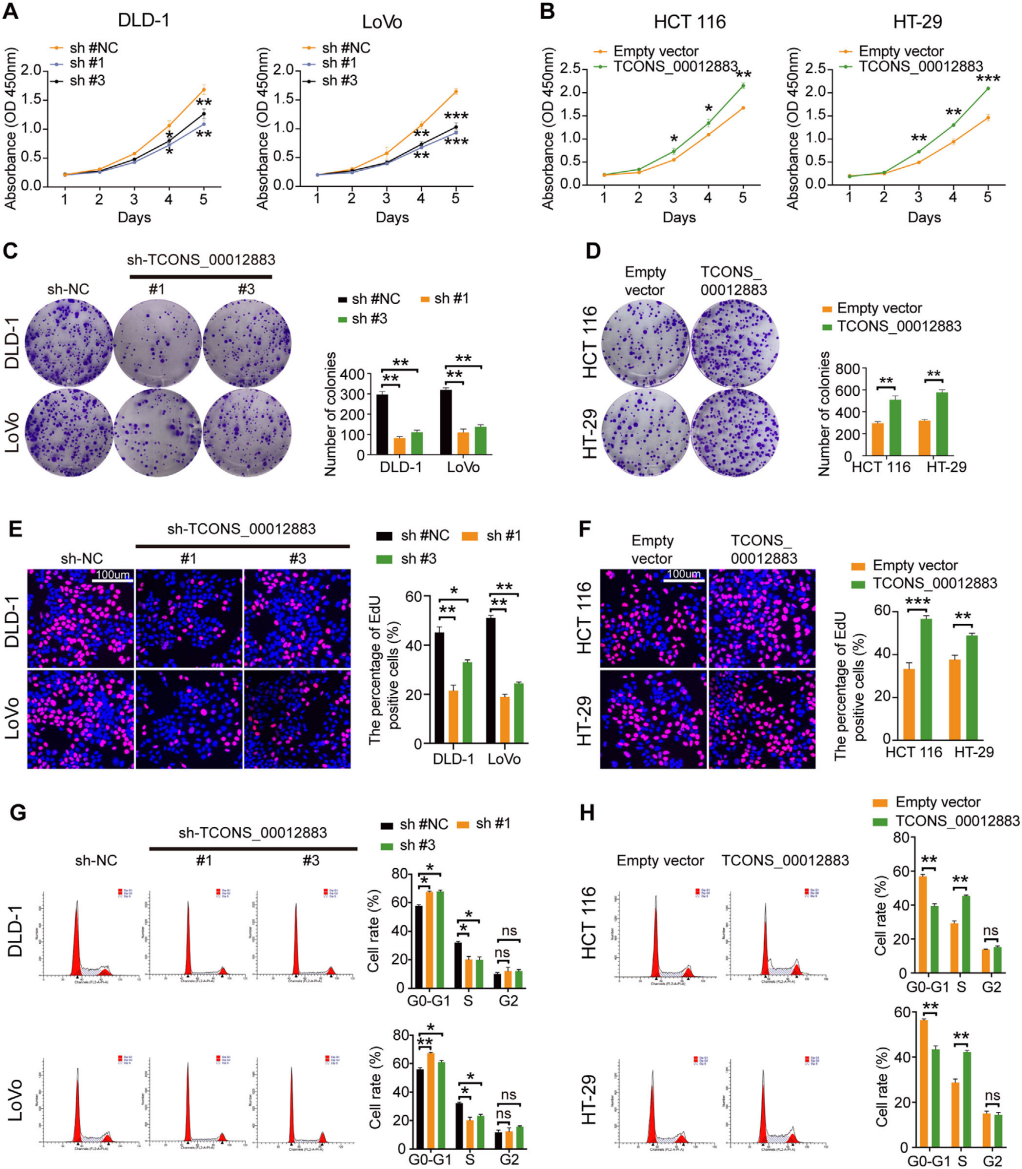
Effects of TCONS_00012883 on CRC cells proliferation *in vitro*. A, B, CCK8 assays were used to determine the viability of CRC cells after knockdown or overexpression of TCONS_00012883. C, D, Colony formation assays were applied to detect the proliferation of sh‐TCONS_00012883‐transfected or TCONS_00012883 overexpression‐transfected CRC cells. DLD‐1 (sh #NC: 296, sh #1: 82, sh #3: 110), LoVo (sh #NC: 320, sh #1: 110, sh #3: 140), HCT 116 (Empty vector: 300, TCONS_00012883: 510), HT‐29 (Empty vector: 320, TCONS_00012883: 590). E, F, EdU staining assays were conducted to verify the proliferation of cells, which transfected with sh‐TCONS_00012883 or overexpression of TCONS_00012883 (scale bar: 100 μm). DLD‐1 (sh #NC: 0.45, sh #1: 0.22, sh #3: 0.32), LoVo (sh #NC: 0.51, sh #1: 0.19, sh #3: 0.24), HCT 116 (Empty vector: 0.34, TCONS_00012883: 0.57), HT‐29 (Empty vector: 0.38, TCONS_00012883: 0.49) G, Flow cytometry showing significant decreases or increases in the proportion of cells in S or G1‐phase, respectively, when TCONS_00012883 was silenced in DLD‐1 and LoVo cells. H, The increase or decrease in the proportion of cell cycle of overexpression transfected HCT 116 and HT‐29 cells was examined by flow cytometry. Data are shown as mean ± SD of three independent experiments, *P < .05, **P < .01, ***P < .001; ns, not significant. DLD‐1 (sh #NC: 58, 33, 9, sh #1: 68, 22, 10, sh #3: 67, 22 11), LoVo (sh #NC: 57, 33, 10, sh #1: 67, 18, 15, sh #3: 60, 24, 16), HCT 116 (Empty vector: 57, 29, 14, TCONS_00012883: 40, 45, 15), HT‐29 (Empty vector: 55, 30, 15, TCONS_00012883: 42, 43, 15)

**FIGURE 3:**
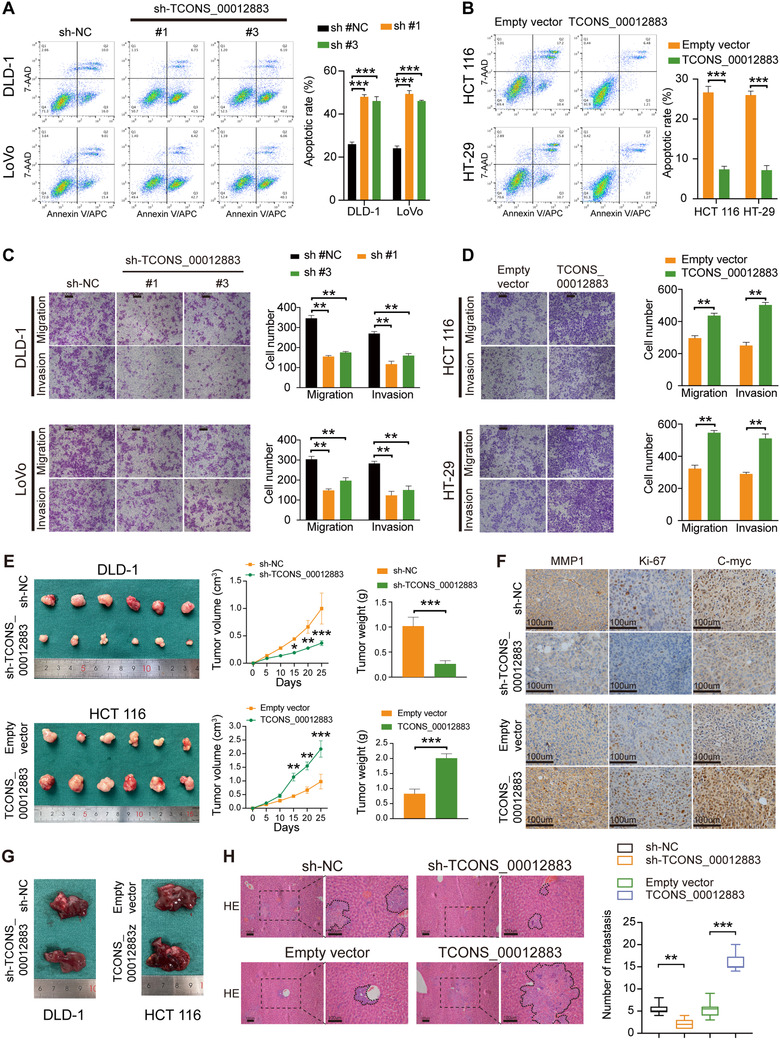
Effects of TCONS_00012883 on CRC cells proliferation, migration, and invasion *in vitro* and *in vivo*. A, B, Cells were treated with 0.5 mM H_2_O_2_ for 4 h. The apoptotic rates (LR + UR) of transfected cells were detected by flow cytometry(Q2 + Q3). LR, early apoptotic cells; UR, terminal apoptotic cells. DLD‐1 (sh #NC: 26, sh #1: 48, sh #3: 46), LoVo (sh #NC: 24, sh #1: 49, sh #3: 45), HCT 116 (Empty vector: 27, TCONS_00012883: 7), HT‐29 (Empty vector: 25, TCONS_00012883: 7). C, D, Transwell assays confirmed that TCONS_00012883 knockdown or overexpression would decrease or increase the migration and invasion of CRC cells, respectively (scale bars: 100 μm). E, Photographs of tumors obtained from the different groups of nude mice transfected with sh‐NC, sh‐TCONS_00012883, Empty vector and TCONS_00012883. Tumors were observed by tumor size and average weight. F, Protein levels of MMP1, Ki‐67, and C‐myc in the tumor samples were determined by IHC (scale bar: 100 μm). G, Representative photograph of liver metastases obtained from nude mice transfected with sh‐NC, sh‐TCONS_00012883, Empty vector and TCONS_00012883. H, HE staining liver metastases in sh‐NC, sh‐TCONS_00012883, Empty vector and TCONS_00012883 (scale bar: 100 μm). Data are shown as mean ± SD of three independent experiments, *P < .05, **P < .01, ***P < .001

Three independent short hairpin RNAs (shRNAs) were transfected against TCONS_00012883 into DLD‐1 and LoVo cells to explore the biological effects of TCONS_00012883 in CRC cell lines, while an overexpressed lentivirus was transfected into HCT 116 and HT‐29 cells, which exhibited higher and lower levels of TCONS_000128833 expression, respectively (Figure 1D). Transfection efficiency was confirmed using qRT‐PCR. As shown in Figure S3A and B, the knockdown efficiency of shRNA#1 and #3 was over 70%, while shRNA#2 did not reach 70%. The stable transfected cells, which contain shRNA#1 and #3, were used for the following assays. In Figure 2A and B, the growth curves were suppressed significantly by the downregulation of TCONS_00012883, while the upregulation of TCONS_000128833 prominently promoted the growth curves of HCT 116 and HT‐29 cells. Colony formation assays similarly indicated that the ability of colony formation of DLD‐1 and LoVo cells was clearly decreased by TCONS_00012883 knockdown, but the overexpression of TCONS_00012883 significantly enhanced the colony forming ability of HCT 116 and HT‐29 cells (Figure 2C, D). Furthermore, the 5‐ethynyl‐2′‐deoxyuridine (EdU) staining assay confirmed that the number of EdU‐positive DLD‐1 and LoVo cells (proliferative cells), which were transfected with shRNAs, was decreased compared with the control group. By contrast, the HCT 116 and HT‐29 cells transfected with overexpressed lentivirus revealed a significant increase compared with the control group (Figure 2E, F). The flow cytometric assays indicated that knockdown of TCONS_00012883 increased the percentage of G0 and G1 phases and decreased S phase populations in CRC cells compared with the control group (Figure 2G). Furthermore, the knockdown of TCONS_00012883 had higher apoptotic rates than those in the control group (Figure 3A). Conversely, the overexpression of TCONS_00012883 promoted the progression of G1‐to‐S phase transformation and suppressed apoptosis (Figures 2H and 3B). Transwell assays revealed that TCONS_00012883 knockdown decreased the ability of cell migration and invasion, while TCONS_00012883 overexpression increased this ability (Figure 3C, D). These findings indicated that TCONS_00012883 behaved as an oncogene to promote CRC cell proliferation and metastasis.

### 3.3 TCONS_00012883 promotes tumor growth and metastasis *in vivo*

As shown in Figure 3E, tumor growth was suppressed by TCONS_00012883 knockdown, with lesser tumor volume and weight than control groups. Furthermore, MMP1 (downstream of TCONS_00012883; Figure 5A‐C), Ki‐67, and C‐myc (proliferation marker) expression levels were detected using IHC staining. The results showed these expression levels were decreased in the sh‐TCONS_00012883 knockdown group and increased in the overexpression group (Figure 3F). A tumor metastasis assay showed that the TCONS_00012883 knockdown group was lessened with small foci in the livers of nude mice. By contrast, TCONS_00012883 overexpression led to a substantial increase in liver metastatic nodules (Figure 3G). Liver tissues were harvested for H&E staining (Figure 3H). Taken together, the results of the *in vivo* experiments strongly suggested that TCONS_00012883 is a novel tumor oncogene with respect to tumor growth and metastasis.

### 3.4 TCONS_00012883 is predominantly localized in the nucleus and interacts with DDX3

**FIGURE 4:**
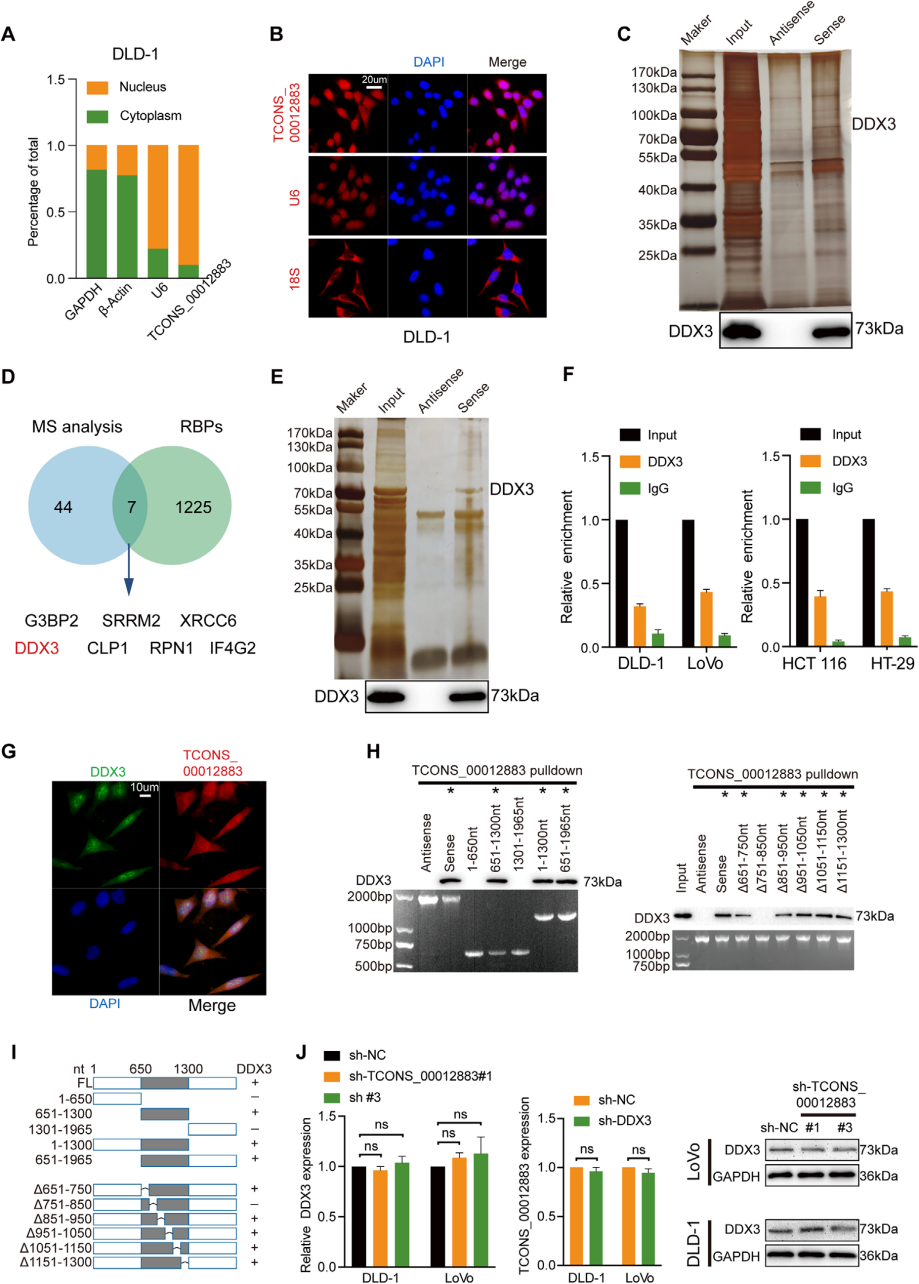
TCONS_00012883 is predominantly localized in the nucleus and interacts with DDX3 protein. A, B, Subcellular fractionation assays and RNA‐FISH confirmed that TCONS_00012883 was predominantly located in the nucleus (scale bar: 20 μm). C‐E, RNA pulldown assay and MS analysis indicated that TCONS_00012883 interacted with DDX3 protein in DLD‐1 cells. F, DDX3 RNA immunoprecipitation (RIP) assay was performed to confirm the interaction between DDX3 and TCONS_00012883. G, Dual RNA‐FISH and immunofluorescence assays (scale bar: 10 μm). H, I, RNA pulldown assay using a series of truncated TCONS_00012883 fragments showed that DDX3 interacted with TCONS_00012883 through its 751‐850 nt sequence. J, Expression of DDX3 was confirmed by qRT‐PCR and western blot in sh‐TCONS_00012883 transfected CRC cell lines. Expression of TCONS_00012883 was verified in sh‐DDX3 transfected CRC cell lines by qRT‐PCR. Data are shown as mean ± SD of three independent experiments, *P < .05, **P < .01, ***P < .001; ns, not significant

Subcellular fractionation assays and RNA‐FISH revealed that TCONS_00012883 was predominantly localized in the nucleus (Figure 4A, B; Figure S4A, B). Next, biotin‐labeled RNA pulldown was conducted to identify the protein partner of TCONS_00012883 by using biotinylated sense TCONS_00012883 and biotinylated antisense TCONS_00012883 transcript (Figure 4C). A mass spectrometry assay revealed 51 differential proteins between the sense and antisense TCONS_00012883 transcript pulldown groups in DLD‐1 cells (Table S2). Afterward, overlapped differential proteins with RBPs (Table S3) and seven proteins were found to be potential partners (Figure 4D). Considering TCONS_00012883 was mainly localized in the nucleus, nucleoprotein possibly could interact with TCONS_00012883. Biotin‐labeled RNA pulldown assay was then performed with nucleoprotein in DLD‐1 cells. The results showed that a protein with 70 kDa could be a potential RBP (Figure 4E). Among the seven differential proteins, DDX3, but not others, could be the protein partner of TCONS_00012883. WB showed that the DDX3 could be detected in the input group and TCONS_00012883 pulldown products, but not in the antisense pulldown products (Figure 4C‐E). The RIP assay showed that TCONS_00012883 could be enriched by DDX3, but not antisense TCONS_00012883 (Figure 4F). Dual RNA‐FISH and IF assays also showed that DDX3 protein and TCONS_00012883 were mainly located in the same location (Figure 4G). Furthermore, to map the TCONS_00012883 functional motifs corresponding to DDX3 binding, RNA pulldown assay was conducted using a series of truncated TCONS_00012883 fragments. Five transcript nucleotide sequences (1‐650 nt, 651‐1300 nt, 1301‐1965 nt, 1‐1300 nt, and 651‐1965 nt) were designed and carried out to identify DDX3 binding region through RNA pulldown assays. The results revealed that the 651‐1300 nt of TCONS_00012883 was the potential binding region of DDX3 (Figure 4H). To further clarify the precise sequences of 651‐1300 nt that binds to DDX3 protein, we constructed a delete mutation (Δ) of the 651‐1300 nt region (Δ651‐750 nt, Δ751‐850 nt, Δ851‐950 nt, Δ951‐1050 nt, Δ1051‐1150 nt, Δ1151‐1250 nt, and Δ1251‐1300 nt). RNA pulldown assays confirmed that the TCONS_00012883 sequence deletion of the 751‐850 nt region did not detect DDX3 protein in the pulldown products (Figure 4H, I). After TCONS_00012883 was knocked down, the level of DDX3 expression did not change. In contrast, the level of TCONS_00012883 expression was not influenced by DDX3 konckdown (Figure 4J and Figure S2C). These findings suggested that TCONS_00012883 interacted with DDX3 protein through its 751‐850 nt sequence.

### 3.5 TCONS_00012883 promotes CRC progression by upregulating the target genes, MMP1

**FIGURE 5:**
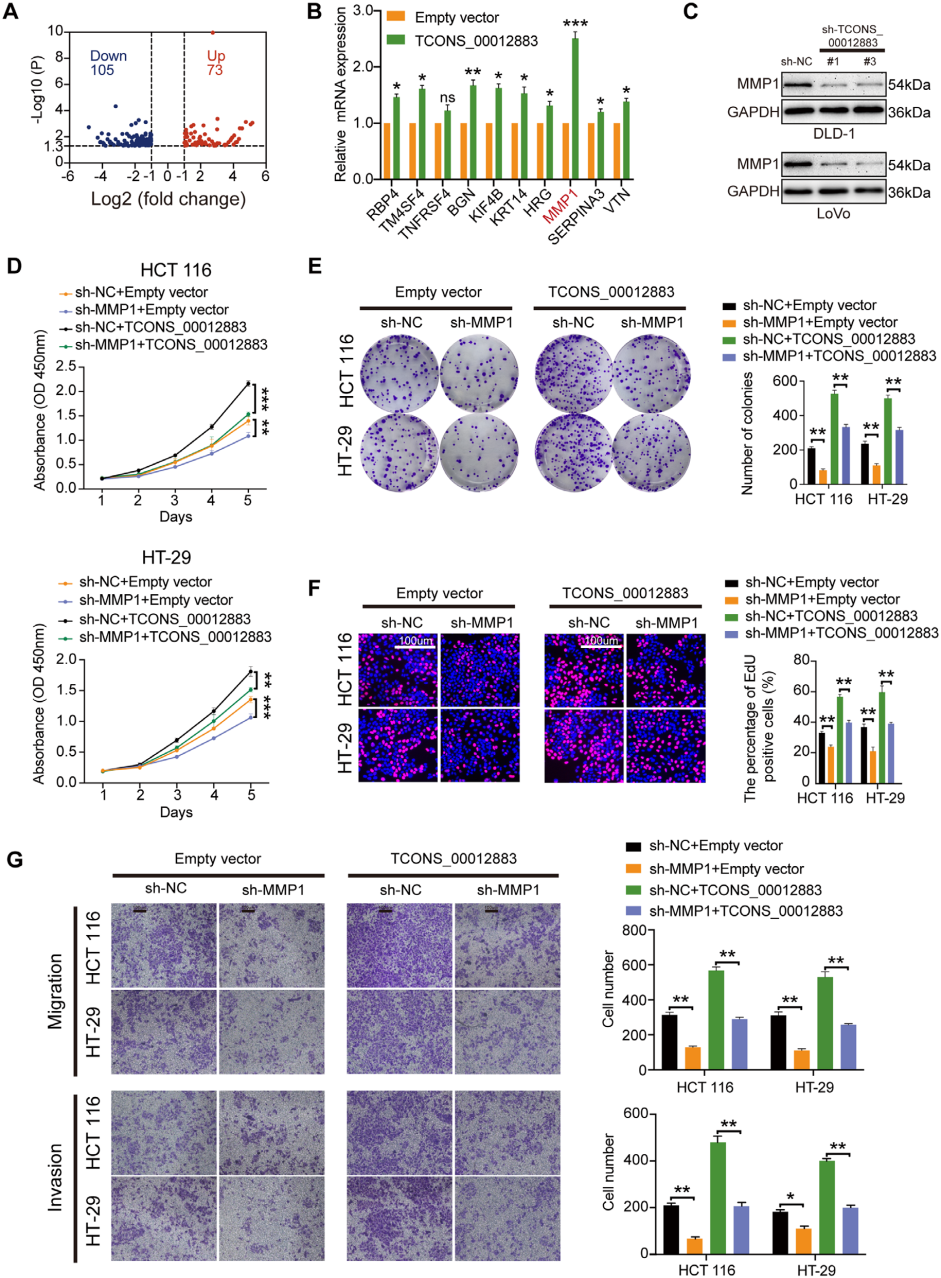
TCONS_00012883 promotes CRC progression by upregulating MMP1. A, RNA‐seq analysis was conducted in 3 pairs HCT 116 cells, which transfected with TCONS_00012883 and Empty vector and indicated that 73 genes upregulated, while 105 genes downregulated. (Fold change > 1 and *P* < .05). B, 10 cancer‐associated genes in upregulated group were detected by qRT‐PCR. C, Western blot analysis of MMP1 after knockdown of TCONS_00012883. D‐G, CCK‐8, colony formation, EdU, and Transwell assay were carried out in cells transfected with sh‐NC+Empty vector, sh‐MMP1+Empty vector, sh‐NC+TCONS_00012883, and sh‐MMP1+TCONS_00012883 (scale bar: 100 μm). Data are shown as mean ± SD of three independent experiments, *P < .05, **P < .01, ***P < .001; ns, not significant

**FIGURE 6:**
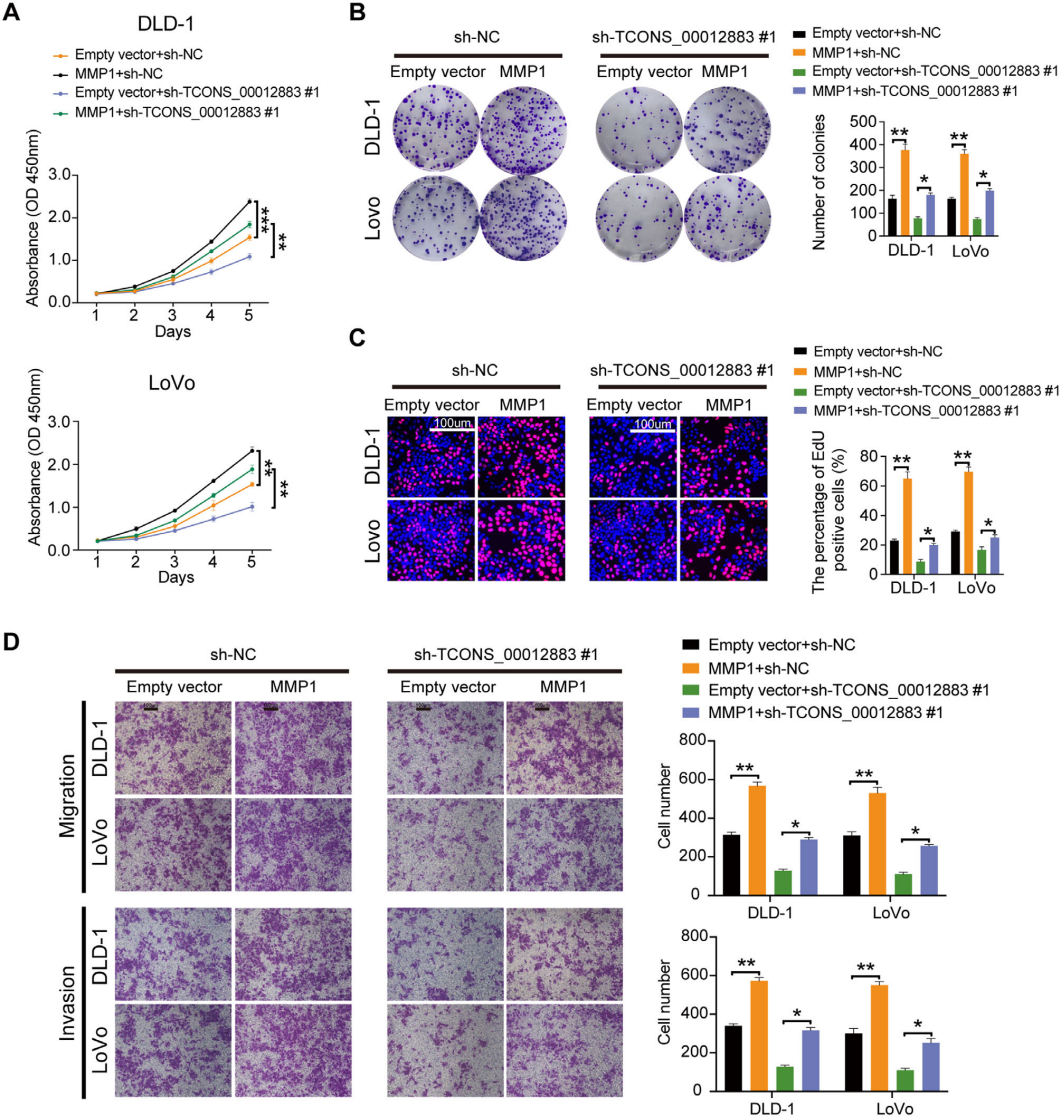
MMP1 promotes CRC progression and could rescue inhibitory effect of sh‐TCONS_00012883. A‐D, CCK‐8, colony formation, EdU, and Transwell assay were carried out in cells transfected with Empty vector+sh‐NC, MMP1+sh‐NC, Empty vector+sh‐TCONS_00012883, and MMP1+sh‐TCONS_00012883 (scale bar: 100 μm). Data are shown as mean ± SD of three independent experiments, *P < .05, **P < .01, ***P < .001

RNA‐seq was performed in HCT 116 cells transfected with overexpressed TCONS_00012883 and control. Among all differentially expressed genes, 73 genes were upregulated and 105 genes were downregulated (Figure 5A). Top‐rank cancer‐associated genes were selected and the level of expression in cells transfected with overexpressed of TCONS_00012883 was confirmed via qRT‐PCR (Figure 5B). Given that MMP1 was changed significantly at the largest, the MMP1 protein level was detected; this level was significantly decreased by TCONS_00012883 knockdown (Figure 5C). Thus, TCONS_00012883 was hypothesized to promote CRC progression by influencing MMP1 expression. MMP1 shRNAs (sh‐MMP1) and negative control (sh‐NC) were transfected into HCT 116 and HT‐29 cells to investigate the oncogenic role of MMP1 in CRC. Transfection efficiency was examined at the mRNA levels (Figure S2D). As shown in Figure 5D, sh‐MMP1 remarkably inhibited cell growth curves, while MMP1 overexpression could promote CRC cell growth curves (Figure 6A). Similar results were also found in colony formation, EdU, and transwell assays (Figures 5E‐G and 6B‐D). Rescue experiments indicated that the cotransfection group (TCONS_00012883 overexpression and sh‐MMP1) could reverse the growth curves caused by TCONS_00012883 overexpression (Figure 5D). The opposite results were noted in the group of sh‐TCONS_00012883 and MMP1 overexpression (Figure 6A). Colony formation, EdU, and transwell assays also yielded similar results (Figures 5E‐G and 6B‐D), thus indicating that TCONS_00012883 exerted malignant properties by influencing the expression of MMP1.

### 3.6 TCONS_00012883 regulates MMP1 expression by interacting with DDX3 to mediate the transactivation of YY1

**FIGURE 7:**
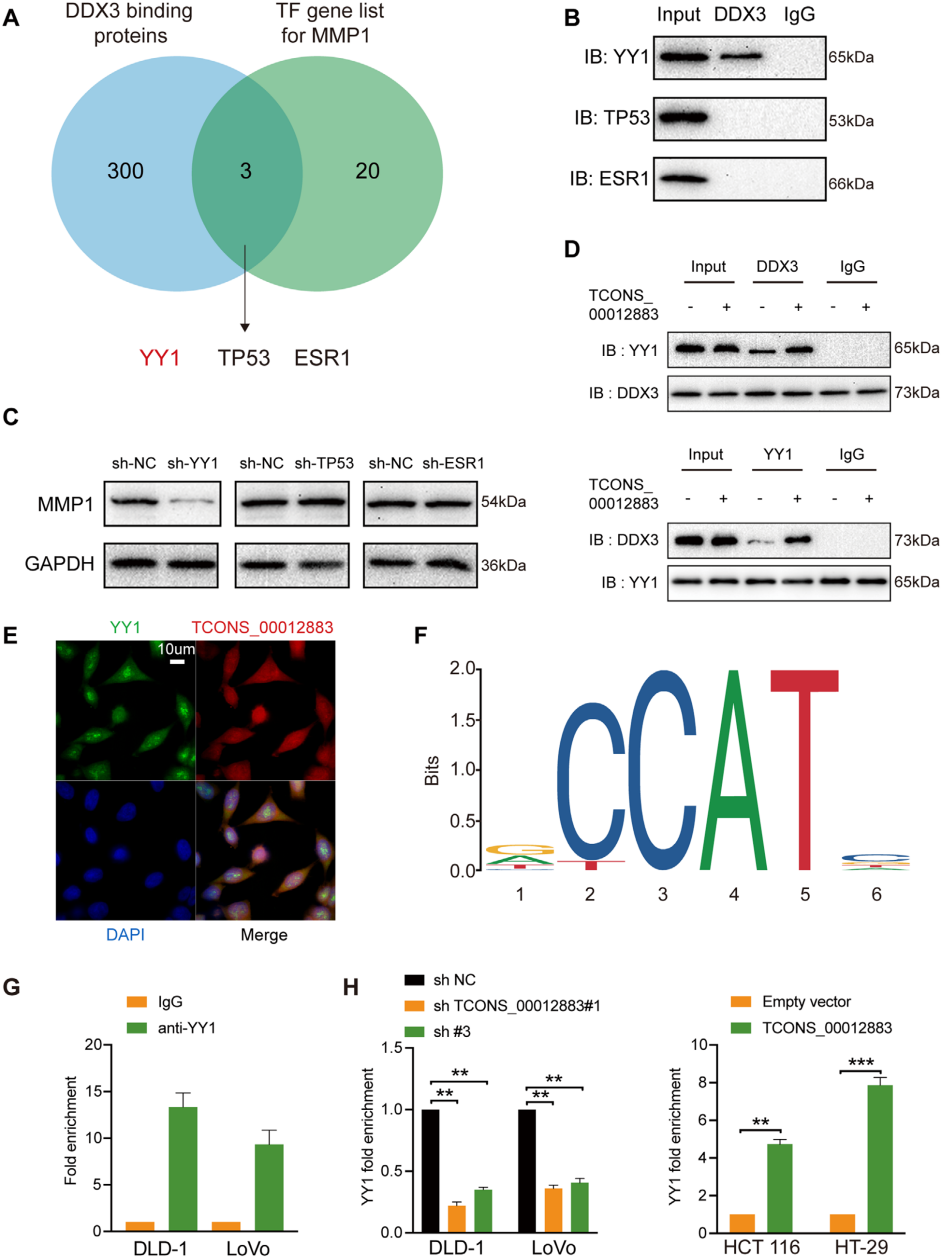
TCONS_00012883 cooperates with DDX3 to mediate MMP1 expression by mediating transactivation of YY1. A, Venn diagram revealed that after overlapping DDX3‐binding proteins from the BioGRID database and transcription factor (TF) list for MMP1, YY1, TP53, and ESR1 were potential transcriptional regulators essential for MMP1. B, Coimmunoprecipitation and western blot assay indicated the interaction between DDX3 and YY1, DDX3 and TP53, DDX3 and ESR1. C, Relative protein levels of MMP1 in DLD‐1 cells, which transfected with sh‐YY1, sh‐TP‐53, sh‐ESR1, and sh‐NC. D, DDX3 and YY1 coimmunoprecipitation and western blot assay were conducted in DLD‐1 cells, which transfected the overexpression of TCONS_00012883 and controls. E, Dual RNA‐FISH and immunofluorescence assays (scale bar: 10 μm). F, The predicted sequence motif using JASPAR database. G, Chromatin immunoprecipitation and qRT‐PCR were conducted in wild‐type DLD‐1 cells and cells that transfected the overexpression of TCONS_00012883 and controls. Data are shown as mean ± SD of three independent experiments, *P < .05, **P < .01, ***P < .001

Previous studies had confirmed that lncRNAs participate in transcriptional regulation through recruiting transcription factors (TFs) in the nucleus. Therefore, we overlapped potential TFs, which may regulate MMP1 expression (PROMO, Table S4) and DDX3‐interacting proteins derived from the BioGRID database (Table S5); the results showed that YY1, TP53, and ESR1 could participate in regulating target gene MMP1 expression (Figure 7A). As shown in Figure 7B, coimmunoprecipitation assay confirmed that YY1, but not TP53 nor ESR1, interacted with DDX3 in DLD‐1 cell lysates. The MMP1 expression in DLD‐1 cells transfected with sh‐YY1, sh‐TP53, and sh‐ESR1, and the negative control (sh‐NC) was detected. The transfection efficiency was confirmed using qRT‐PCR (Figure S3E). Knockdown of YY1 downregulated MMP1 expression compared with the control groups. Similar results could not be replicated in TP53 and ESR1 (Figure 7C). Coimmunoprecipitation assay was then conducted in cells with overexpressed TCONS_00012883 and control group, and the results demonstrated that more YY1 were enriched by DDX3. Similar results were shown in YY1‐co‐IP (Figure 7D). An IF assay also confirmed that TCONS_00012883 and YY1 have a common location (Figure 7E). Moreover, ChIP assay was then performed. The predicted sequence motif using JASPAR database is shown in Figure 7F. The MMP1 in the YY1 group was enriched compared with that in the IgG group (Figure 7G). Another ChIP assay was performed in cells which transfected with sh‐TCONS_00012883 and overexpressed TCONS_00012883. Indeed, TCONS_00012883 silencing attenuated the enrichment of YY1 on MMP1 promoter, while TCONS_00012883 overexpression enhanced the enrichment (Figure 7H). The above data demonstrated that TCONS_00012883, DDX3, and YY1 complex play an important role in regulating MMP1 in CRC cells.

### 3.7 TCONS_00012883/DDX3/YY1/MMP1 axis regulates CRC progression via the PI3K/AKT pathway

**FIGURE 8:**
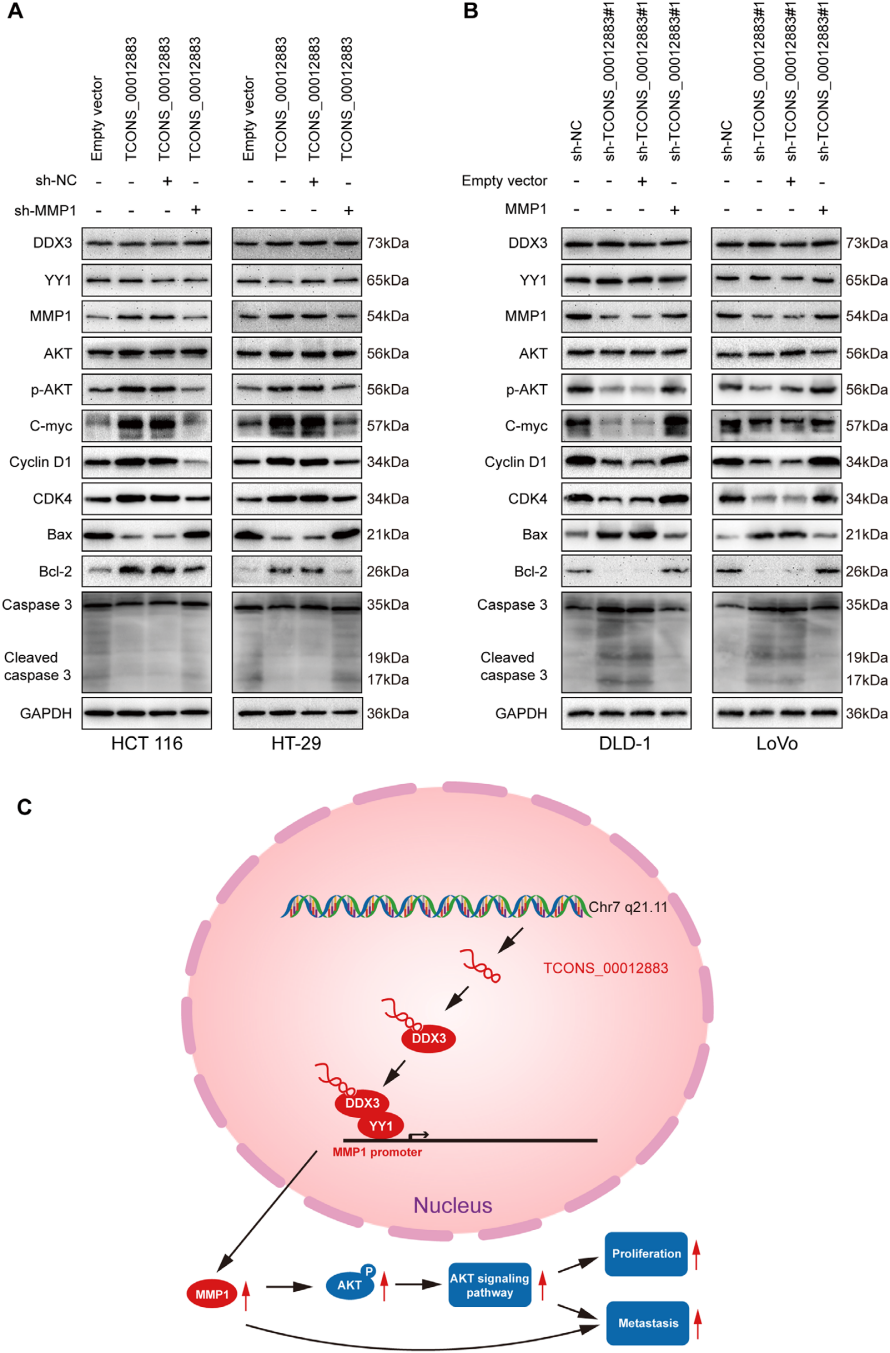
TCONS_00012883 regulates CRC growth and metastasis via the PI3K/AKT pathway. A, Immunoblot analysis of DDX3, YY1, MMP1 total, and phosphorylated Akt, C‐myc, Cyclin D1, CDK4, Bax, Bcl‐2, Caspase 3, Cleaved caspase 3, and GAPDH in CRC cells transfected with Empty vector, TCONS_00012883 and cotransfected groups of sh‐NC and TCONS_00012883, sh‐MMP1 and TCONS_00012883. B, Immunoblot analysis of proteins above in CRC cells transfected with sh‐NC, sh‐TCONS_00012883#1 and cotransfected groups of Empty vector and sh‐TCONS_00012883#1, MMP1 and sh‐TCONS_00012883#1. C, Concise model of TCONS_00012883 in regulating CRC progression. TCONS_00012883 specifically bound to DDX3, increasing DDX3's ability to recruit more transcription factors YY1 to the promoter of MMP1. After transactivation of YY1, MMP1 expression was increased, leading to a regulation of associated module downstream in colorectal cancer

As shown in Figure 8A, the overexpression of TCONS_00012883 greatly increased the levels of MMP1, p‐AKT, C‐myc, Cyclin D1, CDK4, and Bcl‐2 proteins and negatively regulated Bax and Cleaved caspase 3 proteins. The downregulation of TCONS_00012883 generated the opposite results (Figure 8B). In the cotransfected group, the TCONS_00012883‐promoted effect was rescued by MMP1 knockdown (Figure 8A). Opposite results occurred in the sh‐TCONS_0012883 and MMP1 overexpression cotransfected group (Figure 8B). These results confirmed that the TCONS_00012883/DDX3/YY1/MMP1 axis regulates CRC growth and metastasis via the PI3K/AKT pathway.

## 4 DISCUSSION

Accumulating evidence indicates that lncRNAs play a crucial role in human diseases, especially in various cancers.^17^, ^18^, ^19^, ^20^, ^21^, ^22^, ^23^, ^24^, ^25^, ^26^, ^27^, ^28^ LncRNAs participate in almost all cellular process of cancer and serve as critical clinical biomarkers for the diagnosis and prognosis of malignant tumors.^13^, ^29^, ^30^ In the current study, a novel lncRNA, TCONS_00012883, was first identified upregulated in tumor tissues and CRC cell lines. CPAT predicted that TCONS_00012883 has a very low coding potential. There are many reasons for genes upregulation, such as gene mutation or epigenetic changes. In gene mutation, amplification^31^ could cause gene upregulation. In epigenetic changes, transcriptional modification, such as methylation^32^ and TF,^33^ could also cause gene upregulation. Literature review revealed that the pattern of expression and biological functions of TCONS_00012883 in cancer have not been established. Unfortunately, we did not explore the origin of upregulated TCONS_00012883 in our study. We will explore this origin in our future study. Retrospective analysis showed that upregulated TCONS_00012883 was correlated with tumor size, TNM staging system, tumor stage, lymph node metastasis, and distant metastasis. Moreover, increased TCONS_00012883 had shorter OS and PFS time in patients with CRC. Functional experiments demonstrated that upregulated TCONS_00012883 promoted CRC cells proliferation and metastasis, whereas knockdown of TCONS_00012883 suppressed CRC progression. These findings confirmed that TCONS_00012883 can serve as an oncogene in the CRC process.

Previous studies revealed that lncRNAs have various roles depending on their subcellular location.^11^ For example, cytoplasmic lncRNAs could regulate mRNA stability or translation by binding to miRNA binding sites on protein‐coding messengers.^34^ Many nuclear lncRNAs could interact with RBPs and participate in transcriptional and post‐transcriptional regulation. Wang et al confirmed that lncRNA EPIC1 interacts with MYC and promotes cancer progression.^35^ In our study, FISH and subcellular fraction analyses indicated that TCONS_00012883 was mainly localized to the nucleus. Similarly, we found that DDX3 protein was the protein partner of TCONS_00012883 in CRC cells.

DDX3 is a ubiquitous enzyme and belongs to the member of the large DEAD‐box protein family. It has ATP‐dependent RNA helicase activity^36^ and plays an important role in RNA metabolism, such as transcriptional regulation,^37^ mRNP assembly, pre‐mRNA splicing, mRNA export,^37^, ^38^ and regulation of protein‐RNA interactions. Recent studies have confirmed that DDX3 participates in various pathways during cancer progress. For example, DDX3 could interact with CK1e and promote the phosphorylation of Dvl, which subsequently activated WNT/β‐catenin signaling.^39^ DDX3 has been reported to regulate the promoter activity of p21.^37^ In addition, DDX3 promotes the stabilization and nuclear accumulation of p53.^40^ In this study, we demonstrate that DDX3, as a transcriptional regulator, functions as a cofactor to facilitate the transactivation of YY1, which is a TF predicted by Bioinformatic tools (PROMO^41^ and BioGRID database^42^).

YY1 is upregulated in many types of cancers and exerts its oncogenic effects through initiating, activating, or repressing the transcription of target genes.^43^, ^44^ For example, YY1 activated the transcription of lnc00637 to promote the proliferation of breast cancer.^45^ In CRC, YY1 induced the upregulation of ARAP1‐AS1 to promote CRC cells migration and invasion.^46^ In our study, YY1 could activate the transcription of MMP1 to further activate the PI3K/AKT pathway.^47^ Our results indicate that TCONS_00012883 binds the DDX3 protein to increase its interaction with YY1, resulting in the transactivation of YY1 and transcription of MMP1.

However, we confirmed that knockdown of TCONS_00012883 did not influence the DDX3 level. Conversely, the knockdown of DDX3 also did not influence TCONS_00012883 expression. In this case, a series of RNA pulldown assays confirmed that TCONS_00012883 interacted with DDX3 protein through its 751‐850 nt sequences. Unfortunately, we did not further study the specific binding sites on DDX3 protein. We believe that this sequence will be a key target. We suppose that TCONS_00012883 could change the DDX3 protein structure through its 751‐850 nt sequences and the binding site in DDX3 protein to facilitate its interaction with YY1. Regularly, the special sequences may be competitive therapeutic targets of some molecules or medicines. For example, Yang et al confirmed that circ‐CTNNB1 binds the Ia domain of DDX3 protein to increase its interaction with YY1, resulting in the transactivation of YY1. Also, Yang et al designed a cell‐penetrating peptide, named DIP‐13, targeting the Ia domain of DDX3, and they confirmed this peptide could suppress the oncogenic function of circ‐CTNNB1.^48^ However, we did not confirmed that whether there were peptides that could regulate tha oncogenic function of TCONS_00012883. We only confirmed that DDX3 could recruit YY1, and TCONS_00012883 could regulate this recruitment. This hypothesis needs to be verified by subsequent experiments. The underlying mechanisms need further investigation.

## 5 CONCLUSIONS

In summary, for the first time, we identified that TCONS_00012883 is upregulated in tumor tissues and associated with poor prognosis in CRC. TCONS_00012883 could promote the tumor growth and aggressiveness of cancer cells and activate the PI3K/AKT pathway through cooperating with DDX3 to facilitate the transactivation of YY1 and transcriptional alteration of MMP1. Thus, TCONS_00012883 may act as a major role in diagnosis and therapy of CRC.

## CONFLICT OF INTEREST

The authors declared no competing interest exists.

## AUTHOR CONTRIBUTIONS

PY, YFF, and YMS generated the hypothesis and designed the experiments. PY and JL performed experiments. CFP and YQT performed the animal experiments. PY, RRC, WP, JHZ, LW, and QOG interpreted the data. PY wrote the manuscript. YMS, YFF, and JWT supervised the overall research, secured funding, and interpreted results.

## AVAILABILITY OF DATA AND MATERIALS

The datasets used in the current study are available from the corresponding author on reasonable request.

## Supporting information

Figure S1. Levels of seven differentially expressed lncRNAs expression in 24 pairs of tumor tissues with matched normal tissues. Data are presented as the mean ± SD. *P < .05, **P < .01, and ***P < .001, ****P < .0001.

**Figure S2**. Relative expression of TCONS_00012883 in CRC patients with distant metastasis or not. ns, not significant.

**Figure S3**. Transfection efficiency of TCONS_00012883, DDX3, MMP1, YY1, TP53, and ESR1. A, B, Transfection efficiency of knockdown and overexpression of TCONS_00012883 was confirmed using qRT‐PCR in CRC cell lines. C, Transfection efficiency of knockdown of DDX3 was confirmed using qRT‐PCR in CRC cell lines. D, Transfection efficiency of knockdown and overexpression of MMP1 was confirmed using qRT‐PCR in CRC cell lines. E, Transfection efficiency of knockdown of YY1, TP53, and ESR1 was confirmed using qRT‐PCR in CRC cell lines. Data are presented as the mean ± SD. *P < .05, **P < .01, and ***P < .001, ****P < .0001.

**Figure S4**. TCONS_00012883 was predominantly located in the nucleus. A, B, RNA‐FISH and subcellular fractionation assays confirmed that TCONS_00012883 was predominantly located in the nucleus (scale bar: 20 μm).

**Figure S5**. The expression of TCONS_00012883 in CRC, gastric cancer, and pancreatic cancer. Red :TCONS_00012883, blue: DAPI (scale bar: 20 μm).

**Table S1** Correlation between TCONS_00012883 expression and clinicopathological characteristics of CRC patients

**Table S2** Mass spectrometry assay revealed 51 differential proteins

**Table S3** RNA‐binding proteins (RBPs)

**Table S4** TFs gene list for MMP1 with maximum matrix dissimilarity rate 0% from PROMO

**Table S5** DDX3‐interacting proteins from BioGRID database

**Table S6** Sequences of primers and shRNAs

**Table S7** List of primary antibodies used in the study

**Table S8** The raw data for qRT‐PCR in 200 pairs of tumor tissues than in matched normal tissues
