# Supplementary figures and images for: TCONS_00012883 promotes proliferation and metastasis via DDX3/YY1/MMP1/PI3K‐AKT axis in colorectal cancer
Source: Clin Transl Med. 2020 Oct 14;10(6):e211. doi: 10.1002/ctm2.211 (PMC7568852; doi:10.1002/ctm2.211)

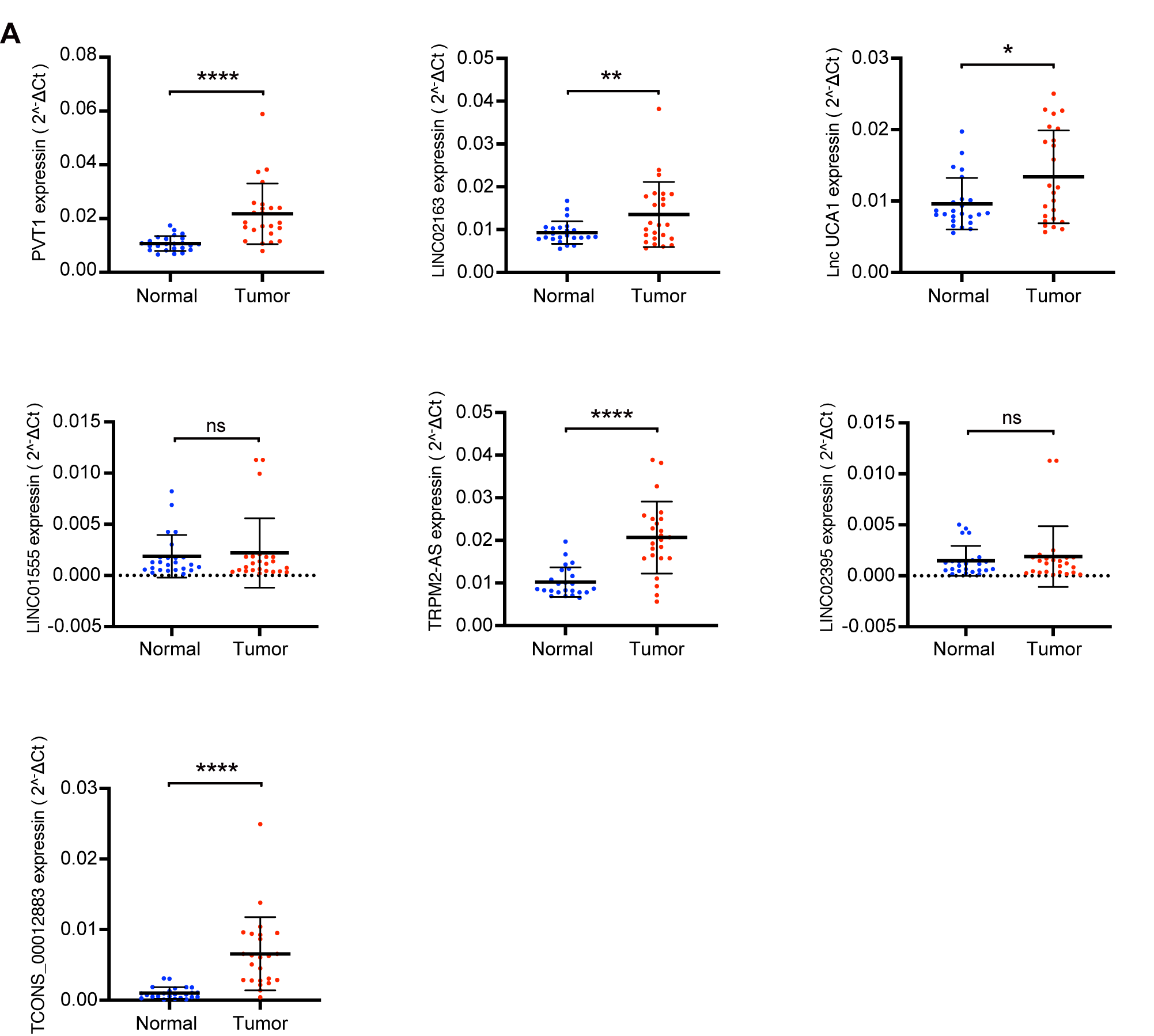

Supplement: Supplementary file 1 — Figure S1. Levels of seven differentially expressed lncRNAs expression in 24 pairs of tumor tissues with matched normal tissues. Data are presented as the mean ± SD. *P < .05, **P < .01, and ***P < .001, ****P < .0001. [file CTM2-10-e211-s001.tif]

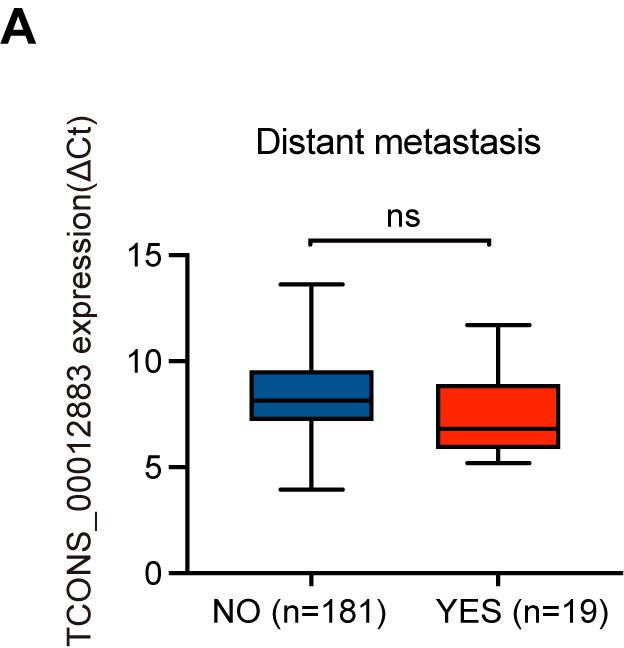

Supplement: Supplementary file 2 — Figure S2. Relative expression of TCONS_00012883 in CRC patients with distant metastasis or not. ns, not significant. [file CTM2-10-e211-s002.tif]

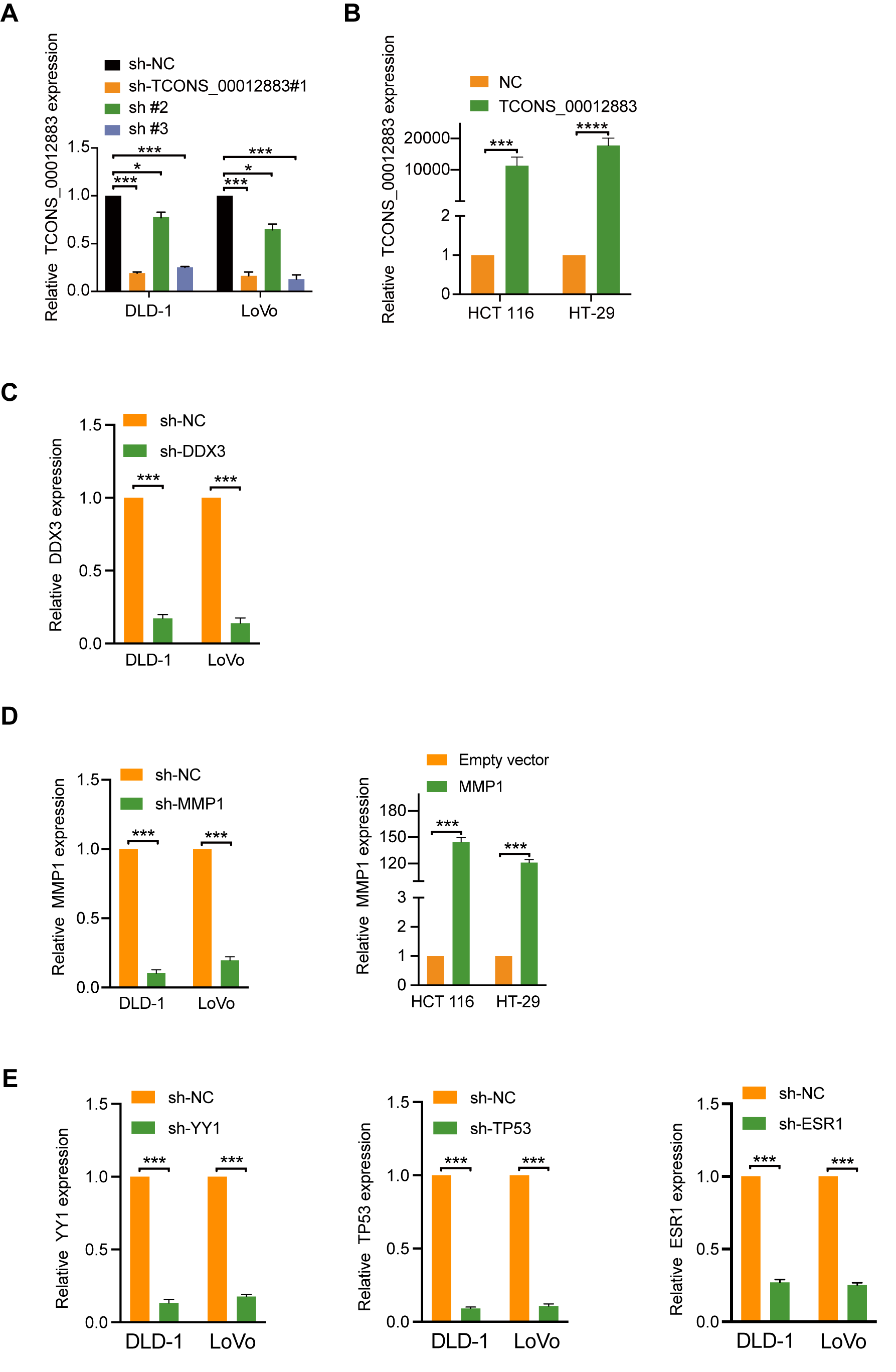

Supplement: Supplementary file 3 — Figure S3. Transfection efficiency of TCONS_00012883, DDX3, MMP1, YY1, TP53, and ESR1. A, B, Transfection efficiency of knockdown and overexpression of TCONS_00012883 was confirmed using qRT‐PCR in CRC cell lines. C, Transfection efficiency of knockdown of DDX3 was confirmed using qRT‐PCR in CRC cell lines. D, Transfection efficiency of knockdown and overexpression of MMP1 was confirmed using qRT‐PCR in CRC cell lines. E, Transfection efficiency of knockdown of YY1, TP53, and ESR1 was confirmed using qRT‐PCR in CRC cell lines. Data are presented as the mean ± SD. *P < .05, **P < .01, and ***P < .001, ****P < .0001. [file CTM2-10-e211-s003.tif]

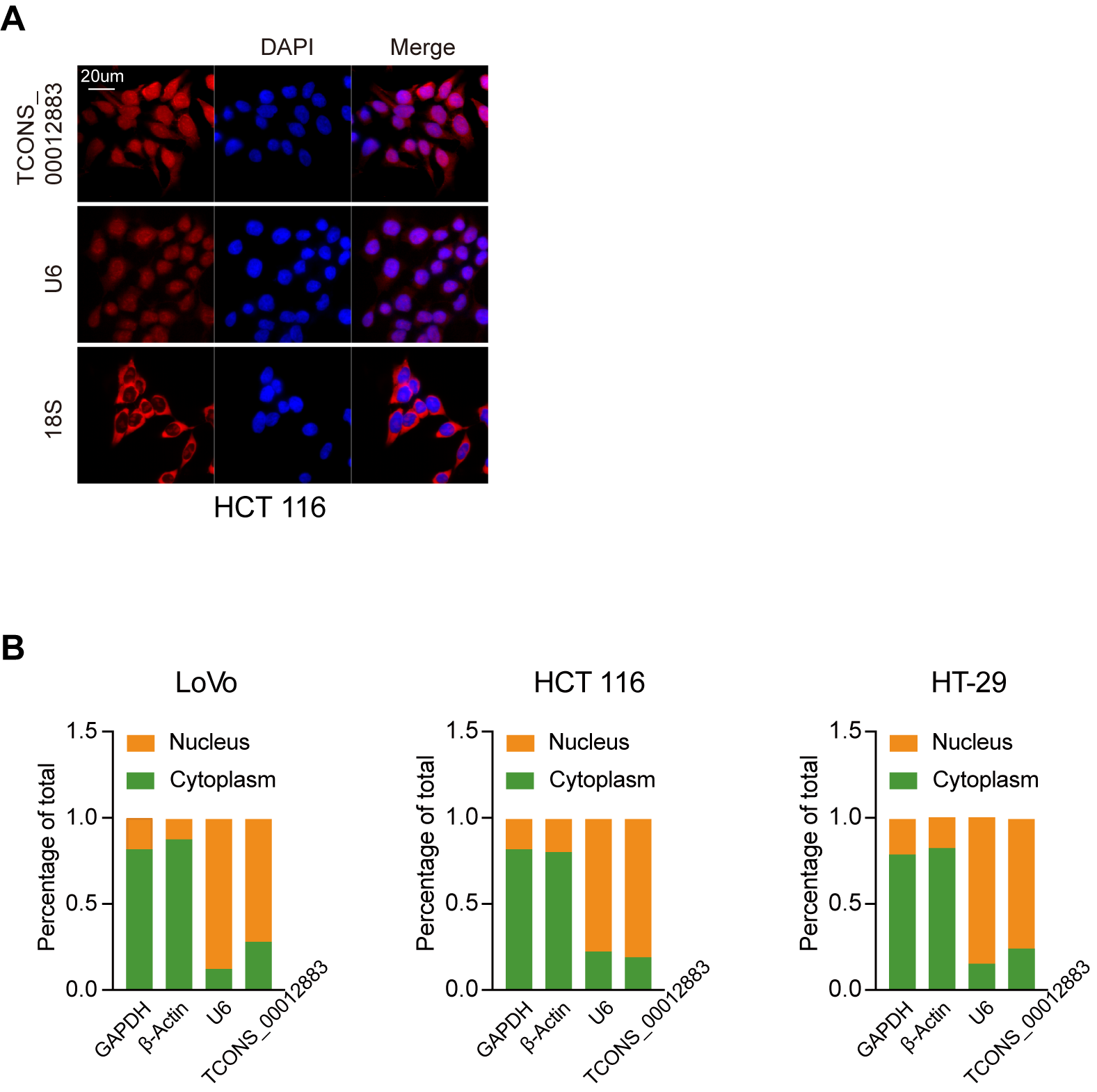

Supplement: Supplementary file 4 — Figure S4. TCONS_00012883 was predominantly located in the nucleus. A, B, RNA‐FISH and subcellular fractionation assays confirmed that TCONS_00012883 was predominantly located in the nucleus (scale bar: 20 μm). [file CTM2-10-e211-s004.tif]

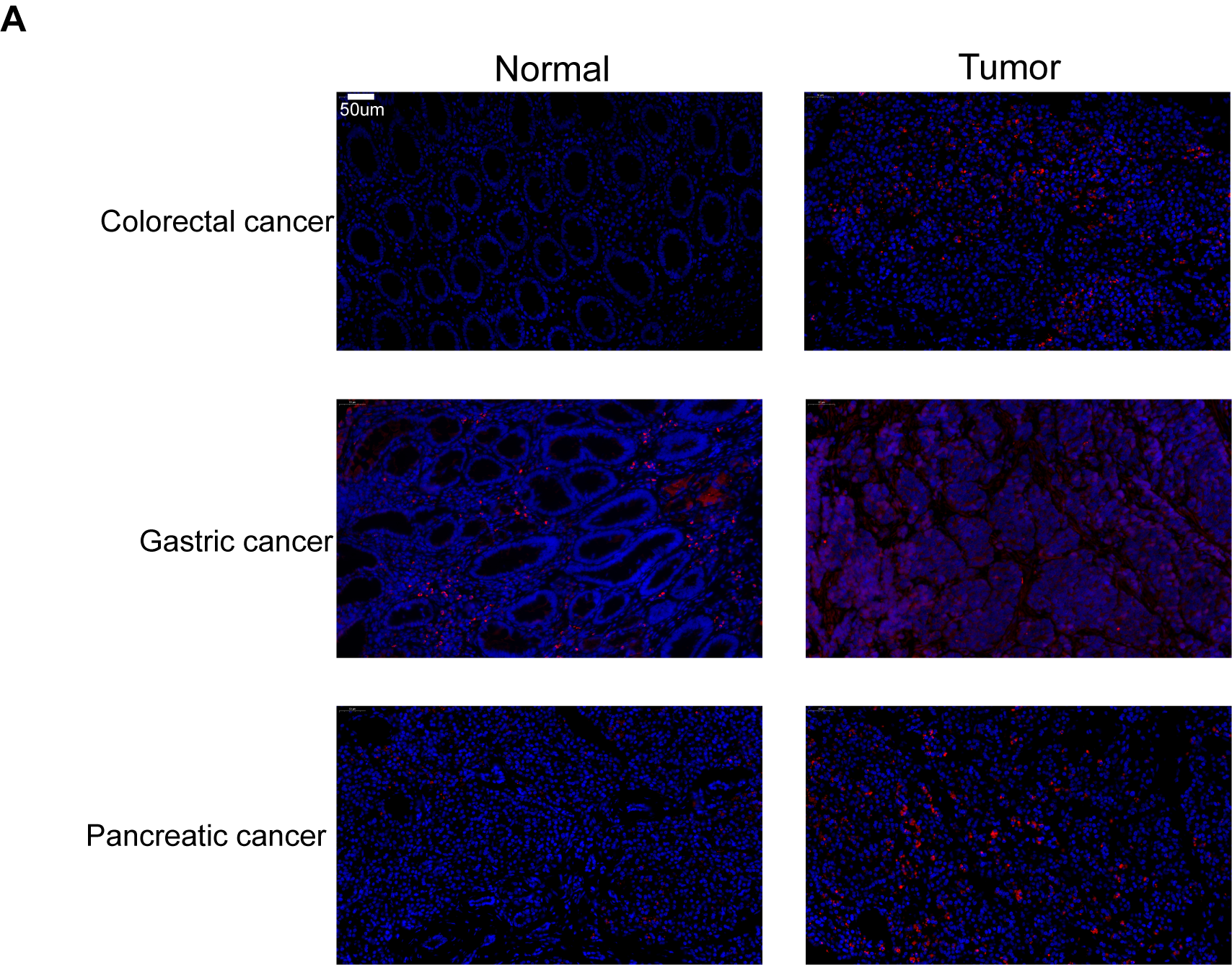

Supplement: Supplementary file 5 — Figure S5. The expression of TCONS_00012883 in CRC, gastric cancer, and pancreatic cancer. Red :TCONS_00012883, blue: DAPI (scale bar: 20 μm). [file CTM2-10-e211-s005.tif]
