# Supplementary material for: TCONS_00012883 promotes proliferation and metastasis via DDX3/YY1/MMP1/PI3K‐AKT axis in colorectal cancer
Source: Clin Transl Med. 2020 Oct 14;10(6):e211. doi: 10.1002/ctm2.211 (PMC7568852; doi:10.1002/ctm2.211)
Supplement: Supplementary file 6 — Table S1 Correlation between TCONS_00012883 expression and clinicopathological characteristics of CRC patients [file CTM2-10-e211-s006.docx]

| **Table S1 Relevance analysis of TCONS_00012883 expression in CRC patients.** | | | | |
| --- | --- | --- | --- | --- |
| **Varible** | **All patients** | **TCONS_00012883** | | **P value** |
|  |  | **High** | **Low** |  |
| All Cases | 200 | 100 | 100 |  |
| Age (years) |  |  |  |  |
| <60 | 67 | 35 | 32 | 0.653 |
| ≥60 | 133 | 65 | 68 |  |
| Gender |  |  |  |  |
| Male | 120 | 64 | 56 | 0.248 |
| Female | 80 | 36 | 44 |  |
| Tumor size (cm) |  |  |  |  |
| <5 | 71 | 25 | 46 | **0.002** |
| ≥5 | 129 | 75 | 54 |  |
| TNM staging system |  |  |  |  |
| T1 + T2 | 35 | 10 | 25 | **0.005** |
| T3 + T4 | 165 | 90 | 75 |  |
| Tumor stage |  |  |  |  |
| Stage I+II | 77 | 30 | 47 | **0.014** |
| Stage III+IV | 123 | 70 | 53 |  |
| Lymph node metastasis |  |  |  |  |
| No | 88 | 35 | 53 | **0.011** |
| Yes | 112 | 65 | 47 |  |
| Distant metastasis |  |  |  |  |
| No | 181 | 86 | 95 | **0.030** |
| Yes | 19 | 14 | 5 |  |
| CEA (ng/ml) |  |  |  |  |
| <5 | 102 | 52 | 50 | 0.777 |
| ≥5 | 98 | 48 | 50 |  |

NOTE: TNM tumour node metastasis. CEA carcinoembryonic antige

P ≤ 0.05 was considered signiﬁcant. The bold type represents P values smaller than 0.05
