# Supplementary material for: TCONS_00012883 promotes proliferation and metastasis via DDX3/YY1/MMP1/PI3K‐AKT axis in colorectal cancer
Source: Clin Transl Med. 2020 Oct 14;10(6):e211. doi: 10.1002/ctm2.211 (PMC7568852; doi:10.1002/ctm2.211)
Supplement: Supplementary file 7 — Table S2 Mass spectrometry assay revealed 51 differential proteins [file CTM2-10-e211-s007.docx]

| **Table S2** | | | |
| --- | --- | --- | --- |
| Mass spectrometry assay revealed 51 differential proteins | | | |
| Gene | Protein_ID | Gene | Protein_ID |
| ZC3H11A | sp\|O75152\|ZC11A_HUMAN | PFKL | sp\|P17858\|PFKAL_HUMAN |
| IRS1 | sp\|P35568\|IRS1_HUMAN | SLC25A13 | sp\|Q9UJS0\|CMC2_HUMAN |
| DDX3X | sp\|O00571\|DDX3X_HUMAN | RPN1 | sp\|P04843\|RPN1_HUMAN |
| HNRNPA1L2 | sp\|Q32P51\|RA1L2_HUMAN | XRCC6 | sp\|P12956\|XRCC6_HUMAN |
| SLC25A6 | sp\|P12236\|ADT3_HUMAN | CEP126 | sp\|Q9P2H0\|CE126_HUMAN |
| SLC25A4 | sp\|P12235\|ADT1_HUMAN | RPL7 | sp\|P18124\|RL7_HUMAN |
| DNAJA3 | sp\|Q96EY1\|DNJA3_HUMAN | G3BP2 | sp\|Q9UN86\|G3BP2_HUMAN |
| RPL36AL | sp\|Q969Q0\|RL36L_HUMAN | EIF4G2 | sp\|P78344\|IF4G2_HUMAN |
| ZG16B | sp\|Q96DA0\|ZG16B_HUMAN | SLC25A22 | sp\|Q9H936\|GHC1_HUMAN |
| HSPA5 | sp\|P11021\|BIP_HUMAN | ARHGAP33 | sp\|O14559\|RHG33_HUMAN |
| SLC25A1 | sp\|P53007\|TXTP_HUMAN | RARS | sp\|P54136\|SYRC_HUMAN |
| FAU | sp\|P62861\|RS30_HUMAN | RPL38 | sp\|P63173\|RL38_HUMAN |
| UNC13B | sp\|O14795\|UN13B_HUMAN | DAZAP1 | sp\|Q96EP5\|DAZP1_HUMAN |
| CLP1 | sp\|Q92989\|CLP1_HUMAN | APOBEC3B | sp\|Q9UH17\|ABC3B_HUMAN |
| MAP6 | sp\|Q96JE9\|MAP6_HUMAN | KRT6A | sp\|P02538\|K2C6A_HUMAN |
| RPL21 | sp\|P46778\|RL21_HUMAN | KRT77 | sp\|Q7Z794\|K2C1B_HUMAN |
| NUDT21 | sp\|O43809\|CPSF5_HUMAN | TRIM21 | sp\|P19474\|RO52_HUMAN |
| TMEM33 | sp\|P57088\|TMM33_HUMAN | RPL10L | sp\|Q96L21\|RL10L_HUMAN |
| OXNAD1 | sp\|Q96HP4\|OXND1_HUMAN | KDELR1 | sp\|P24390\|ERD21_HUMAN |
| RBBP7 | sp\|Q16576\|RBBP7_HUMAN | IMPDH2 | sp\|P12268\|IMDH2_HUMAN |
| SNRPD2 | sp\|P62316\|SMD2_HUMAN | SLC3A2 | sp\|P08195\|4F2_HUMAN |
| SSR3 | sp\|Q9UNL2\|SSRG_HUMAN | SRRM2 | sp\|Q9UQ35\|SRRM2_HUMAN |
| ANLN | sp\|Q9NQW6\|ANLN_HUMAN | RPL18A | sp\|Q02543\|RL18A_HUMAN |
| TUBA1C | sp\|Q9BQE3\|TBA1C_HUMAN | CKAP5 | sp\|Q14008\|CKAP5_HUMAN |
| CHD6 | sp\|Q8TD26\|CHD6_HUMAN | DYSF | sp\|O75923\|DYSF_HUMAN |
| ACACA | sp\|Q13085\|ACACA_HUMAN |  |  |
