# Supplementary material for: TCONS_00012883 promotes proliferation and metastasis via DDX3/YY1/MMP1/PI3K‐AKT axis in colorectal cancer
Source: Clin Transl Med. 2020 Oct 14;10(6):e211. doi: 10.1002/ctm2.211 (PMC7568852; doi:10.1002/ctm2.211)
Supplement: Supplementary file 9 — Table S4 TFs gene list for MMP1 with maximum matrix dissimilarity rate 0% from PROMO [file CTM2-10-e211-s009.docx]

| **Table S4** | |
| --- | --- |
| **Maximum matrix dissimilarity rate：0%** | **ID** |
| TFs gene list for MMP1 | CEBPB |
|  | TFAP2A |
|  | NR3C1 |
|  | STAT4 |
|  | GTF2I |
|  | GATA1 |
|  | TBP |
|  | YY1 |
|  | XBP1 |
|  | GR-alpha |
|  | IRF2 |
|  | FOXA1 |
|  | PAX5 |
|  | ESR1 |
|  | NR3C1 |
|  | FOXP3 |
|  | ENKTF-1 |
|  | PGR |
|  | PR A |
|  | HOXD9 |
|  | HOXD10 |
|  | TP53 |
|  | JUN |
