# Supplementary material for: TCONS_00012883 promotes proliferation and metastasis via DDX3/YY1/MMP1/PI3K‐AKT axis in colorectal cancer
Source: Clin Transl Med. 2020 Oct 14;10(6):e211. doi: 10.1002/ctm2.211 (PMC7568852; doi:10.1002/ctm2.211)
Supplement: Supplementary file 12 — Table S7 List of primary antibodies used in the study [file CTM2-10-e211-s012.docx]

| **Table S7** | | |
| --- | --- | --- |
| **Antigens** | **Manufacturer** | **Application** |
| DDX3 | Santa Cruz Biotechnology : sc-365768 | 1:100 for WB; 1:50 for IF |
| YY1 | Cell Signaling Technology : #63227 | 1:1000 for WB; 1:200 for IF |
| TP53 | Abcam : ab26 | 1:1000 for WB |
| ESR1 | Cell Signaling Technology : #13258 | 1:1000 for WB |
| MMP1 | Abcam : ab137332 | 1:1000 for WB;1:100 for IHC |
| AKT | Abcam : ab8805 | 1:500 for WB |
| P-AKT | Abcam : ab38449 | 1:500 for WB |
| C-myc | Abcam : ab32072 | 1:1000 for WB; 1:200 for IHC |
| Cyclin D1 | Abcam : ab134175 | 1:5000 for WB |
| CDK4 | Abcam : ab108357 | 1:1000 for WB |
| Caspase 3 | Cell Signaling Technology : #9662 | 1:1000 for WB |
| Bcl-2 | Abcam : ab32124 | 1:1000 for WB |
| Bax | Abcam : ab32503 | 1:1000 for WB |
| GAPDH | Abcam : ab9485 | 1:2500 for WB |
| Ki-67 | Abcam : ab15580 | 1:500 for IHC |
